# The Dry Treatment of Chancroids

**Published:** 1891-11

**Authors:** 


### THE DRY TREATMENT OF CHANCROIDS.

It is generally conceded that if chancroidal ulcers can be kept very dry, a great step has been taken toward their rapid healing. With this view, the following procedure has been used to some extent in the surgical divisions of Bellevue Hospital, New York : A small roll of absorbent cotton about one-half an inch in diameter and long enough to surround the penis just behind the corona, is put in that position after the prepuce has been well retracted. A rubber thread band is slipped over this ring of cotton in order to hold it in place. By this means the sulcus behind the glands is obliterated, which is especially liable to retain the secretions, and the prepuce is held back from contact with the ulcerated surface. The cotton absorbs the exudation from those surfaces almost as soon as formed. The dressing is light, is easily handled, and may be renewed as often as needed to keep the parts in a dry condition. In addition to chancroids, herpes preputialis and venereal warts have been found to heal rapidly under the use of this dressing; sometimes no other treatment has been found necessary for these local lesions.—*Canada Record.*

